# *N*-Phenyl­formamide

**DOI:** 10.1107/S1600536809022776

**Published:** 2009-06-20

**Authors:** B. Thimme Gowda, Sabine Foro, Hartmut Fuess

**Affiliations:** a Department of Chemistry, Mangalore University, Mangalagangotri 574 199, Mangalore, India; b Institute of Materials Science, Darmstadt University of Technology, Petersenstrasse 23, D-64287 Darmstadt, Germany

## Abstract

There are two independent mol­ecules in the asymmetric unit of the title compound, C_7_H_7_NO. The conformation of the N—H bond in the structure is *syn* to the C=O bond in one of the mol­ecules and *anti* in the other. In the crystal, mol­ecules are packed into chains diagonally in the *ac* plane *via* N—H⋯O hydrogen bonds.

## Related literature

For related structures, see: Gowda *et al.* (2006 ▶); Brown (1966 ▶). For our study of the effect of ring and side chain substitutions on the crystal structures of aromatic amides, see: Gowda *et al.* (2000 ▶, 2007 ▶, 2009 ▶).   
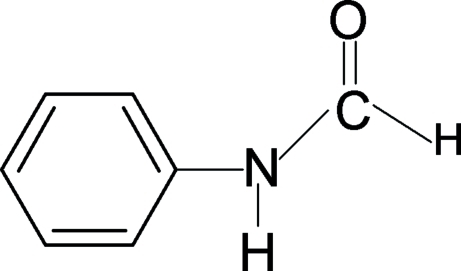


## Experimental

### 

#### Crystal data

C_7_H_7_NO
*M* *_r_* = 121.14
Monoclinic, 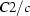

*a* = 30.923 (3) Å
*b* = 6.1737 (6) Å
*c* = 14.814 (1) Å
β = 113.14 (1)°
*V* = 2600.6 (4) Å^3^
*Z* = 16
Mo *K*α radiation
μ = 0.08 mm^−1^
*T* = 298 K
0.48 × 0.44 × 0.40 mm

#### Data collection

Oxford Diffraction Xcalibur diffractometer with a Sapphire CCD detector
Absorption correction: multi-scan (*CrysAlis RED*; Oxford Diffraction, 2009 ▶) *T*_min_ = 0.966, *T*_max_ = 0.969
8394 measured reflections
2383 independent reflections
1679 reflections with *I* > 2σ(*I*)
*R*_int_ = 0.016

#### Refinement

*R*[*F* ^2^ > 2σ(*F* ^2^)] = 0.037
*wR*(*F* ^2^) = 0.115
*S* = 1.12
2383 reflections
170 parameters
H atoms treated by a mixture of independent and constrained refinement
Δρ_max_ = 0.11 e Å^−3^
Δρ_min_ = −0.10 e Å^−3^

### 

Data collection: *CrysAlis CCD* (Oxford Diffraction, 2009 ▶); cell refinement: *CrysAlis RED* (Oxford Diffraction, 2009 ▶); data reduction: *CrysAlis RED*; program(s) used to solve structure: *SHELXS97* (Sheldrick, 2008 ▶); program(s) used to refine structure: *SHELXL97* (Sheldrick, 2008 ▶); molecular graphics: *PLATON* (Spek, 2009 ▶); software used to prepare material for publication: *SHELXL97*.

## Supplementary Material

Crystal structure: contains datablocks I, global. DOI: 10.1107/S1600536809022776/rk2151sup1.cif

Structure factors: contains datablocks I. DOI: 10.1107/S1600536809022776/rk2151Isup2.hkl

Additional supplementary materials:  crystallographic information; 3D view; checkCIF report

## Figures and Tables

**Table 1:** Hydrogen-bond geometry (Å, °)

*D*—H⋯*A*	*D*—H	H⋯*A*	*D*⋯*A*	*D*—H⋯*A*
N1—H1*N*⋯O2^i^	0.888 (16)	1.936 (16)	2.8239 (17)	178.1 (14)
N2—H2*N*⋯O1^ii^	0.857 (16)	2.007 (16)	2.8637 (17)	177.0 (14)

Symmetry codes: (i) 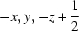; (ii) 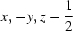.

**Fig. 1.**
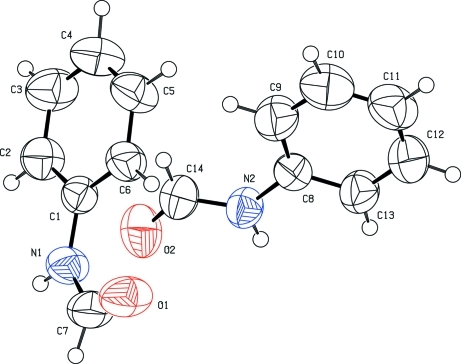
Molecular structure of (I), showing the atom labelling scheme. Displacement ellipsoids are drawn at the 50% probability level. H atoms are represented as a small spheres of arbitrary radii.

**Fig. 2.**
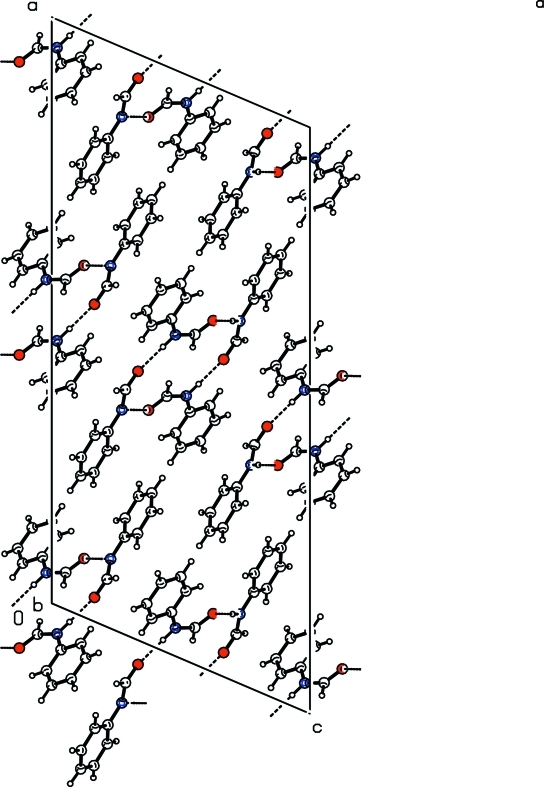
Molecular packing of (I) with hydrogen bonding shown as dashed lines.

## supplementary crystallographic information

### Comment

As part of a study of the effect of ring and side chain substitutions on the crystal structures of aromatic amides (Gowda *et al.*, 2000; 2007; 2009), the structure of *N*–(phenyl)–formamide (I) has been determined. The asymmetric unit contains two independent molecules (Fig. 1). The conformation of the N—H bond is *syn* to the C═O bond in the side chain, in one of the molecules and is *anti* in the other, in contrast to the *anti* conformation observed in *N*–(2,6–dichlorophenyl)–formamide (Gowda *et al.*, 2000 and *N*–(phenyl)–acetamide (Brown *et al.*, 1966). The molecules in (I) are linked through intermolecular N—H···O hydrogen bonding (Tab. 1) and the chains formed diagonally as viewed in the *ac* plane (Fig. 2).

### Experimental

The purity of the commmercial sample (Aldrich Chemicals) was checked by determining its melting point and characterized by recording its infrared and NMR spectra (Gowda *et al.*, 2006). The single crystals used in *X*–ray diffraction studies were grown in ethanol solution by slow evaporation at room temperature.

### Refinement

The H atoms were located in difference map and their positional parameters were refined with C—H = 0.9300 Å with *U*_iso_(H) = 1.2*U*_eq_(C). The N–bonded H atoms refined freely.

### Crystal data

**Table:**

C_7_H_7_NO	*F*(000) = 1024
*M**_r_* = 121.14	*D*_x_ = 1.238 Mg m^−^^3^
Monoclinic, *C*2/*c*	Mo *K*α radiation, λ = 0.71073 Å
Hall symbol: -C 2yc	Cell parameters from 2475 reflections
*a* = 30.923 (3) Å	θ = 2.7–28.2°
*b* = 6.1737 (6) Å	µ = 0.08 mm^−^^1^
*c* = 14.814 (1) Å	*T* = 298 K
β = 113.14 (1)°	Needle, colourless
*V* = 2600.6 (4) Å^3^	0.48 × 0.44 × 0.40 mm
*Z* = 16	

### Data collection

**Table:**

Oxford Diffraction Xcalibur diffractometer with a Sapphire CCD detector	2383 independent reflections
Radiation source: Fine–focus sealed tube	1679 reflections with *I* > 2σ(*I*)
Graphite	*R*_int_ = 0.016
ω and φ scans	θ_max_ = 25.4°, θ_min_ = 2.8°
Absorption correction: multi-scan (*CrysAlis RED*; Oxford Diffraction, 2009)	*h* = −37→36
*T*_min_ = 0.966, *T*_max_ = 0.969	*k* = −7→7
8394 measured reflections	*l* = −17→17

### Refinement

**Table:**

Refinement on *F*^2^	Secondary atom site location: difference Fourier map
Least-squares matrix: full	Hydrogen site location: inferred from neighbouring sites
*R*[*F*^2^ > 2σ(*F*^2^)] = 0.037	H atoms treated by a mixture of independent and constrained refinement
*wR*(*F*^2^) = 0.115	*w* = 1/[σ^2^(*F*_o_^2^) + (0.0651*P*)^2^] where *P* = (*F*_o_^2^ + 2*F*_c_^2^)/3
*S* = 1.12	(Δ/σ)_max_ < 0.001
2383 reflections	Δρ_max_ = 0.11 e Å^−^^3^
170 parameters	Δρ_min_ = −0.10 e Å^−^^3^
0 restraints	Extinction correction: *SHELXL97* (Sheldrick, 2008), Fc^*^=kFc[1+0.001xFc^2^λ^3^/sin(2θ)]^-1/4^
Primary atom site location: structure-invariant direct methods	Extinction coefficient: 0.0028 (7)

### Special details

**Table:**

Experimental. Absorption correction: CrysAlis RED (Oxford Diffraction, 2009); empirical absorption correction using spherical harmonics, implemented in SCALE3 ABSPACK scaling algorithm.
Geometry. All s.u.'s (except the s.u. in the dihedral angle between two l.s. planes) are estimated using the full covariance matrix. The cell s.u.'s are taken into account individually in the estimation of s.u.'s in distances, angles and torsion angles; correlations between s.u.'s in cell parameters are only used when they are defined by crystal symmetry. An approximate (isotropic) treatment of cell s.u.'s is used for estimating s.u.'s involving l.s. planes.
Refinement. Refinement of *F*^2^ against ALL reflections. The weighted *R*–factor *wR* and goodness of fit *S* are based on *F*^2^, conventional *R*–factors *R* are based on *F*, with *F* set to zero for negative *F*^2^. The threshold expression of *F*^2^ > 2σ(*F*^2^) is used only for calculating *R*–factors(gt) *etc*. and is not relevant to the choice of reflections for refinement. *R*–factors based on *F*^2^ are statistically about twice as large as those based on *F*, and *R*–factors based on ALL data will be even larger.

### Fractional atomic coordinates and isotropic or equivalent isotropic displacement parameters (Å^2^)

**Table:**

	*x*	*y*	*z*	*U*_iso_*/*U*_eq_	
O1	0.09999 (4)	0.21410 (18)	0.62537 (8)	0.0921 (4)	
N1	0.04841 (4)	0.34831 (19)	0.47961 (9)	0.0694 (4)	
H1N	0.0202 (6)	0.322 (2)	0.4332 (11)	0.083*	
C1	0.07229 (4)	0.5289 (2)	0.46365 (9)	0.0561 (3)	
C2	0.04734 (5)	0.6657 (3)	0.38753 (10)	0.0727 (4)	
H2	0.0159	0.6367	0.3491	0.087*	
C3	0.06857 (6)	0.8441 (3)	0.36825 (12)	0.0893 (5)	
H3	0.0515	0.9351	0.3164	0.107*	
C4	0.11462 (6)	0.8895 (3)	0.42461 (12)	0.0890 (5)	
H4	0.1289	1.0119	0.4119	0.107*	
C5	0.13948 (6)	0.7535 (3)	0.49972 (12)	0.0828 (5)	
H5	0.1709	0.7834	0.5378	0.099*	
C6	0.11890 (5)	0.5734 (2)	0.51989 (11)	0.0687 (4)	
H6	0.1363	0.4818	0.5712	0.082*	
C7	0.06296 (6)	0.2094 (2)	0.55435 (12)	0.0792 (5)	
H7	0.0426	0.0966	0.5518	0.095*	
O2	0.04140 (4)	0.2563 (2)	0.16582 (9)	0.0996 (4)	
N2	0.11861 (4)	0.1904 (2)	0.22825 (8)	0.0628 (3)	
H2N	0.1131 (5)	0.067 (3)	0.1994 (10)	0.075*	
C8	0.16640 (5)	0.2389 (2)	0.28235 (9)	0.0565 (3)	
C9	0.18179 (6)	0.4299 (3)	0.33258 (13)	0.0862 (5)	
H9	0.1603	0.5369	0.3309	0.103*	
C10	0.22921 (7)	0.4618 (3)	0.38543 (14)	0.1024 (6)	
H10	0.2394	0.5901	0.4202	0.123*	
C11	0.26131 (6)	0.3093 (3)	0.38760 (14)	0.0963 (6)	
H11	0.2932	0.3324	0.4235	0.116*	
C12	0.24605 (6)	0.1230 (3)	0.33668 (13)	0.0919 (5)	
H12	0.2678	0.0184	0.3371	0.110*	
C13	0.19895 (5)	0.0867 (2)	0.28451 (11)	0.0750 (4)	
H13	0.1891	−0.0425	0.2503	0.090*	
C14	0.08175 (6)	0.3109 (3)	0.21441 (12)	0.0771 (4)	
H14	0.0867	0.4471	0.2436	0.093*	

### Atomic displacement parameters (Å^2^)

**Table:**

	*U*^11^	*U*^22^	*U*^33^	*U*^12^	*U*^13^	*U*^23^
O1	0.0813 (8)	0.0791 (7)	0.0881 (8)	−0.0120 (6)	0.0032 (6)	0.0215 (5)
N1	0.0527 (7)	0.0658 (8)	0.0743 (8)	−0.0096 (6)	0.0082 (6)	0.0067 (6)
C1	0.0522 (8)	0.0564 (8)	0.0570 (7)	−0.0001 (6)	0.0186 (6)	−0.0032 (6)
C2	0.0644 (9)	0.0780 (10)	0.0644 (9)	−0.0023 (7)	0.0131 (7)	0.0049 (7)
C3	0.1006 (14)	0.0823 (11)	0.0750 (10)	−0.0058 (10)	0.0236 (9)	0.0207 (9)
C4	0.0993 (14)	0.0849 (12)	0.0826 (11)	−0.0263 (10)	0.0355 (10)	0.0063 (9)
C5	0.0682 (10)	0.0902 (12)	0.0847 (11)	−0.0210 (9)	0.0242 (8)	0.0026 (9)
C6	0.0555 (8)	0.0699 (9)	0.0742 (9)	−0.0033 (7)	0.0184 (7)	0.0063 (7)
C7	0.0714 (10)	0.0648 (9)	0.0897 (11)	−0.0116 (8)	0.0189 (9)	0.0098 (8)
O2	0.0556 (7)	0.1164 (10)	0.1092 (9)	0.0152 (6)	0.0132 (6)	0.0123 (7)
N2	0.0567 (7)	0.0615 (7)	0.0645 (7)	0.0048 (6)	0.0175 (6)	−0.0040 (5)
C8	0.0557 (8)	0.0602 (8)	0.0522 (7)	−0.0013 (6)	0.0197 (6)	0.0026 (6)
C9	0.0790 (12)	0.0746 (10)	0.1005 (12)	−0.0037 (8)	0.0303 (9)	−0.0202 (9)
C10	0.0939 (14)	0.0939 (13)	0.1047 (13)	−0.0311 (11)	0.0233 (11)	−0.0271 (10)
C11	0.0647 (11)	0.1055 (15)	0.0999 (13)	−0.0158 (11)	0.0122 (9)	0.0068 (11)
C12	0.0601 (10)	0.0924 (12)	0.1119 (13)	0.0070 (9)	0.0218 (9)	0.0067 (10)
C13	0.0618 (9)	0.0680 (9)	0.0873 (10)	0.0029 (7)	0.0209 (8)	−0.0064 (7)
C14	0.0667 (11)	0.0739 (10)	0.0865 (11)	0.0132 (8)	0.0256 (9)	0.0104 (8)

### Geometric parameters (Å, °)

**Table:**

O1—C7	1.2132 (17)	O2—C14	1.2178 (18)
N1—C7	1.3314 (19)	N2—C14	1.3082 (19)
N1—C1	1.4076 (17)	N2—C8	1.4089 (17)
N1—H1N	0.888 (16)	N2—H2N	0.857 (16)
C1—C2	1.3771 (19)	C8—C13	1.3684 (19)
C1—C6	1.3797 (18)	C8—C9	1.375 (2)
C2—C3	1.369 (2)	C9—C10	1.378 (2)
C2—H2	0.9300	C9—H9	0.9300
C3—C4	1.367 (2)	C10—C11	1.359 (3)
C3—H3	0.9300	C10—H10	0.9300
C4—C5	1.365 (2)	C11—C12	1.354 (3)
C4—H4	0.9300	C11—H11	0.9300
C5—C6	1.371 (2)	C12—C13	1.373 (2)
C5—H5	0.9300	C12—H12	0.9300
C6—H6	0.9300	C13—H13	0.9300
C7—H7	0.9300	C14—H14	0.9300
			
C7—N1—C1	128.45 (13)	C14—N2—C8	128.51 (14)
C7—N1—H1N	115.8 (10)	C14—N2—H2N	115.9 (10)
C1—N1—H1N	115.7 (10)	C8—N2—H2N	115.6 (10)
C2—C1—C6	119.24 (13)	C13—C8—C9	118.76 (14)
C2—C1—N1	117.46 (12)	C13—C8—N2	117.69 (12)
C6—C1—N1	123.29 (12)	C9—C8—N2	123.55 (13)
C3—C2—C1	120.27 (14)	C8—C9—C10	119.64 (16)
C3—C2—H2	119.9	C8—C9—H9	120.2
C1—C2—H2	119.9	C10—C9—H9	120.2
C4—C3—C2	120.51 (15)	C11—C10—C9	121.22 (17)
C4—C3—H3	119.7	C11—C10—H10	119.4
C2—C3—H3	119.7	C9—C10—H10	119.4
C5—C4—C3	119.30 (15)	C12—C11—C10	118.93 (17)
C5—C4—H4	120.4	C12—C11—H11	120.5
C3—C4—H4	120.4	C10—C11—H11	120.5
C4—C5—C6	121.06 (15)	C11—C12—C13	120.85 (17)
C4—C5—H5	119.5	C11—C12—H12	119.6
C6—C5—H5	119.5	C13—C12—H12	119.6
C5—C6—C1	119.62 (14)	C8—C13—C12	120.59 (15)
C5—C6—H6	120.2	C8—C13—H13	119.7
C1—C6—H6	120.2	C12—C13—H13	119.7
O1—C7—N1	126.94 (14)	O2—C14—N2	124.22 (16)
O1—C7—H7	116.5	O2—C14—H14	117.9
N1—C7—H7	116.5	N2—C14—H14	117.9
			
C7—N1—C1—C2	−171.26 (15)	C14—N2—C8—C13	−178.73 (14)
C7—N1—C1—C6	9.0 (2)	C14—N2—C8—C9	1.3 (2)
C6—C1—C2—C3	−0.2 (2)	C13—C8—C9—C10	−1.4 (2)
N1—C1—C2—C3	180.00 (14)	N2—C8—C9—C10	178.58 (15)
C1—C2—C3—C4	−0.5 (3)	C8—C9—C10—C11	1.1 (3)
C2—C3—C4—C5	0.8 (3)	C9—C10—C11—C12	0.0 (3)
C3—C4—C5—C6	−0.5 (3)	C10—C11—C12—C13	−0.8 (3)
C4—C5—C6—C1	−0.2 (2)	C9—C8—C13—C12	0.7 (2)
C2—C1—C6—C5	0.5 (2)	N2—C8—C13—C12	−179.34 (13)
N1—C1—C6—C5	−179.70 (14)	C11—C12—C13—C8	0.4 (3)
C1—N1—C7—O1	1.5 (3)	C8—N2—C14—O2	179.62 (13)

### Hydrogen-bond geometry (Å, °)

**Table:**

*D*—H···*A*	*D*—H	H···*A*	*D*···*A*	*D*—H···*A*
N1—H1N···O2^i^	0.888 (16)	1.936 (16)	2.8239 (17)	178.1 (14)
N2—H2N···O1^ii^	0.857 (16)	2.007 (16)	2.8637 (17)	177.0 (14)

Symmetry codes: (i) −*x*, *y*, −*z*+1/2; (ii) *x*, −*y*, *z*−1/2.
